# Only Three Fingers Write, but the Whole Brain Works^†^: A High-Density EEG Study Showing Advantages of Drawing Over Typing for Learning

**DOI:** 10.3389/fpsyg.2017.00706

**Published:** 2017-05-09

**Authors:** Audrey L. H. van der Meer, F. R. (Ruud) van der Weel

**Affiliations:** Developmental Neuroscience Laboratory, Department of Psychology, Norwegian University of Science and TechnologyTrondheim, Norway

**Keywords:** educational psychology and R&D, cognitive neuroscience, electroencephalography (EEG), electrophysiology of handwriting, teaching

## Abstract

Are different parts of the brain active when we type on a keyboard as opposed to when we draw visual images on a tablet? Electroencephalogram (EEG) was used in young adults to study brain electrical activity as they were typing or describing in words visually presented Pictionary^TM^ words using a keyboard, or as they were drawing pictures of the same words on a tablet using a stylus. Analyses of temporal spectral evolution (time-dependent amplitude changes) were performed on EEG data recorded with a 256-channel sensor array. We found that when drawing, brain areas in the parietal and occipital regions showed event related desynchronization activity in the theta/alpha range. Existing literature suggests that such oscillatory neuronal activity provides the brain with optimal conditions for learning. When describing the words using the keyboard, upper alpha/beta/gamma range activity in the central and frontal brain regions were observed, especially during the ideation phase. However, since this activity was highly synchronized, its relation to learning remains unclear. We concluded that because of the benefits for sensory-motor integration and learning, traditional handwritten notes are preferably combined with visualizations (e.g., small drawings, shapes, arrows, symbols) to facilitate and optimize learning.

## Introduction

The general effectiveness of notetaking in educational settings is well-documented, but the evidence mainly stems from a time when laptop use in classrooms was not very common. Previous research has focused on how encoding affects learning (e.g., Kiewra, 1989). The encoding hypothesis proposes that the processing that occurs during notetaking enhances recall and retention. Notetaking can be generative (e.g., summarizing, reframing, paraphrasing) or non-generative (i.e., verbatim transcribing). Verbatim notetaking typically involves relatively shallow cognitive processing (Craik and Lockhart, 1972; Kiewra, 1985). Greater encoding benefits have been observed the more deeply information is processed during notetaking (DiVesta and Gray, 1973). Studies have shown that non-verbatim notetaking leads to better performance than verbatim notetaking, especially on conceptual items (Aiken et al., 1975; Bretzing and Kulhavy, 1979; Slotte and Lonka, 1999; Igo et al., 2005). Traditional laptop use, using the keyboard, promotes verbatim transcription of lecture content because most students can type much faster than they can write (Brown, 1988). Thus, typing may undermine the encoding benefits seen in past notetaking studies.

A recent study by Mueller and Oppenheimer (2014) addressed potential differences in laptop versus longhand notetaking. College students watched 30-min TED Talks on specialist topics that were not common knowledge. They received either laptops or notebooks, and were instructed to take notes using whatever strategy they normally used. Immediately after watching the lecture, they had to answer factual-recall questions and conceptual-application questions. The results indicated that both types of note takers performed equally well on questions that involved recalling facts, while longhand note takers performed significantly better on the conceptual questions. The authors suggested that laptop note takers might engage in less extensive cognitive processing than longhand note takers. From this study it seems that using traditional pen and paper is preferable over traditional laptop use, using the keyboard, when taking notes. However, it is hard to imagine that people will actually be switching back to using pen and paper. Yet, there are several new stylus technologies available on the market today, allowing students to have an electronic record of their notes, and at the same time encouraging them to process the information coming in through the fingers, eyes and ears, rather than mindlessly transcribing it.

The present study was carried out with this in mind. Based on the cognitive processing dichotomy of shallow versus deep encoding, we designed an experiment to investigate electrophysiological differences in brain activity that could explain the differences underlying traditional (keyboard) and more modern (stylus technology) writing. We based our task on the popular family game of Pictionary^TM^ involving three different conditions: (a) typing visual words on a keyboard involving shallow encoding; (b) describing visual words on a keyboard involving deep encoding; and (c) drawing visual words with a stylus involving deep encoding. It was investigated which parts of the brain were active during these three conditions and how the different parts of the brain were communicating with each other. It should be noted that in this study we compared typing on a keyboard with drawing a picture on a tablet with a pen, under the assumption that handwriting and drawing with a pen, in general, involve similar brain activity (see Potgieser et al., 2015). Furthermore, Penketh (2011) argued that handwriting and drawing involve similarly complex skills in translating three-dimensional shapes onto a flat plane. In both, there is the need for visual processing and sensory integration (vision, touch), and this is combined with manual dexterity (skilled hand movement) required to put pen to paper, including eye-hand coordination.

## Materials and Methods

### Participants

We recruited 20 students (12 females) between 21 and 25 years from our local University campus (NTNU, Trondheim, Norway). Seventeen provided sufficient artifact-free data for the analyses. Participants were given the Edinburgh Handedness Inventory (Oldfield, 1971) to determine dominant hand use. We only accepted right-handed participants to the study with a laterality quotient > +0.6. All participants gave their informed written consent and had the liberty to withdraw from the experiment at any time. According to the local and national ethical guidelines and regulations, a full review and approval of the study was not required. Participants were after the experiment rewarded with a US $20 cinema ticket.

### Experimental Stimuli and Paradigm

**FIGURE 1:**
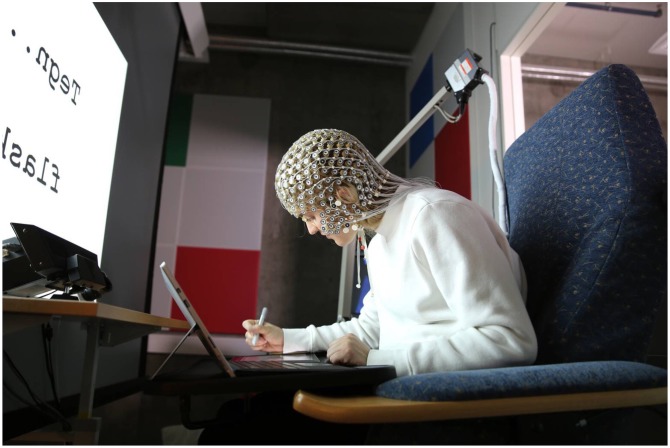
**Experimental set-up with a participant wearing the Geodesic Sensor Net.** On the large screen right in front of the participant Pictionary^TM^ words were projected that either had to be typed up, described in words, or drawn as pictures on one of the two laptop tablets in front of the participant using the keyboard (for typing and describing) or the stylus (for drawing).

**FIGURE 2:**
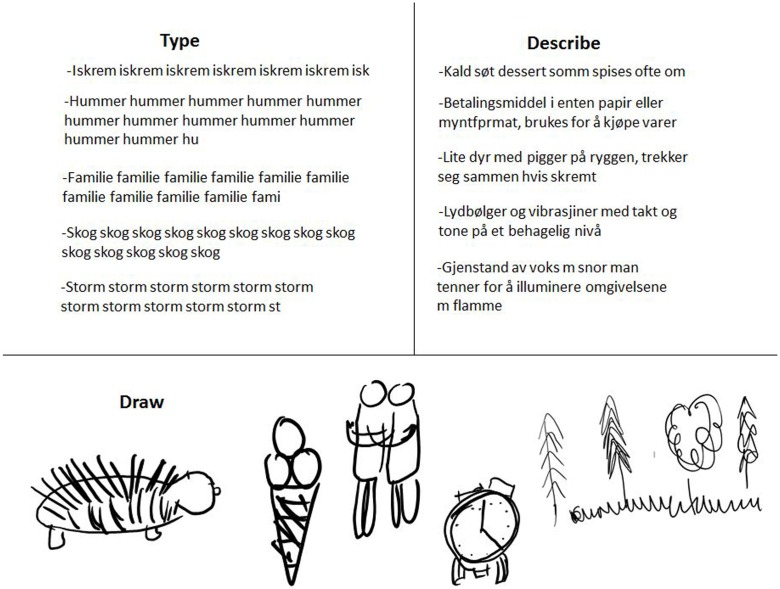
**Illustrative sample of participants’ responses to visually presented Pictionary^TM^ words: typed up and described in words using the laptop tablet keyboard, and drawn as visual images on the laptop tablet with a stylus**.

An ASK M2 projector^1^ was used to project the target words onto a rectangular display (108 cm wide, 70.5 cm high) at a distance of 80 cm in front of the participant (see **Figure 1**). Psychological software tool, E-prime 1.2^2^, was used to generate 20 different Pictionary^TM^ words from the medium difficulty section of the Pictionary^TM^ application “Game Words”^3^. The 20 selected words were presented three times each in a random order. For each trial, participants were instructed to either (a) type the word repetitively separated by a single space using their right index finger on the laptop tablet keyboard, (b) type a description of the word using their right index finger on the laptop tablet keyboard, and (c) draw a picture of the word using their right hand with the stylus on a second identical laptop tablet (for examples of participants’ responses, see **Figure 2**). There were two laptop tablets used in the experiment to minimize unnecessary movement in between trials that could cause artifacts in the data. One laptop tablet was attached to a keyboard and the other one came with a stylus. The laptop tablets were made available by Microsoft, Europe for the duration of the experiment. We used two identical Microsoft Surface Pro 4 laptop tablets^4^; 256 GB/Intel Core i5 – 8 GB RAM with Type Cover and Surface-pen attached. Laptop data produced by the participants were stored in Microsoft OneNote for offline analyses.

### EEG Data Acquisition

Electroencephalography (EEG) activity was recorded with a Geodesic Sensor Net 200^5^ (Tucker, 1993) consisting of an array of 256 sensors that were evenly distributed on the head of the participant (see **Figure 1**). A high-input EGI amplifier ensured amplification of signals at maximum impedance of 50 kΩ as recommended for an optimal signal-to-noise ratio (Picton et al., 2000; Ferree et al., 2001). Net Station software on a separate computer recorded amplified EEG signals at a sampling rate of 250 Hz. Recorded data were afterward stored for off-line analyses.

### Procedure

On arrival at the laboratory, participants received the necessary information for signing the consent form, and the Edinburgh Handedness Inventory was administered. The participant’s head circumference was measured and the correct net size was selected. After soaking the net in a saline electrolyte solution to ensure optimal electrical conductivity, it was slightly dried with a towel before it was mounted on the participant’s head. The participant was then led into a dimly lit experimental room that was separated with a transparent window from a control room containing the data acquisition computers. The participant sat in front of the screen in a comfortable adjustable chair with a fold away desk (see **Figure 1**). The net was connected to the amplifier and the impedance of the electrodes was checked. If necessary, contact of electrodes was improved by adding saline electrolyte to the electrodes or simply adjusting their position.

The experimental session began immediately after the participant’s electrode impedance was approved. After a few initial practice trials, target words were presented in a random sequential order on the screen for a fixed number of trials, 60 per participant: 20 Pictionary^TM^ words in the type condition; the same 20 words in the describe condition; and the same 20 words in the draw condition. Each word appeared on screen for 25 s and sound signals indicated the start and end of each trial. Participants were instructed to start typing or drawing as soon as the word appeared on screen and to move as little as possible during the 5 s recording time to avoid artifacts caused by eye, head, and body movements. Data acquisition was carried out in one block and lasted for about 45 min. However, word presentation was paused in the event of a participant indicating a need to the control room.

### Pre-analyses

Electroencephalography raw data were analyzed with Brain Electrical Source Analysis (BESA^6^) research software version 6.1. As an initial pre-processing step, recordings were segmented with Net Station software and exported as raw files with the appropriate auxiliary files attached. Averaging epoch was from -300 to 5000 ms at a baseline definition of -300 to 0 ms. The notch filter was set at 50 Hz to remove line interference from the data. A low cut-off filter was set at 1.6 Hz to remove slow drift in the data, while a high cut-off was set at 75 Hz. Artifact-contaminated channels and epochs resulting from head or body movements were excluded from further analyses or their signals estimated using spherical spline interpolation (Perrin et al., 1989; Picton et al., 2000). Three participants with 10% of channels defined as bad were excluded from further analysis. In scanning for artifacts, threshold values for gradient and low signal were set at 75 and 0.1 μV, respectively, while maximum amplitude was at 200 μV. Manual artifact correction designed to separate brain activity from artifacts using spatial filters was applied to correct for physiological artifacts caused by blinking or eye movements (Berg and Scherg, 1994; Ille et al., 2002; Fujioka et al., 2011). The mean number of accepted trials for all participants was 56 (*SD* = 3) more or less evenly distributed over the three experimental conditions.

### Time-Frequency Analyses in Brain Space

**FIGURE 3:**
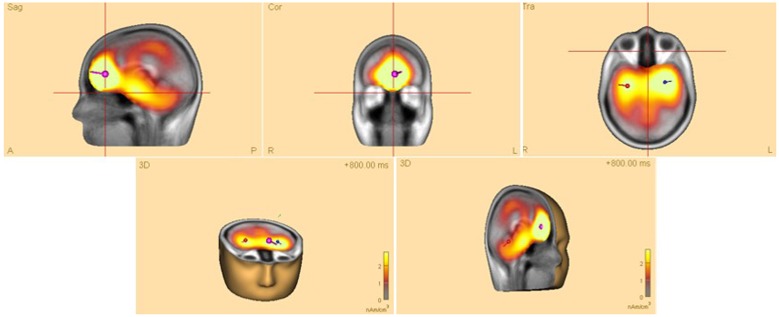
**Head model of a typical (female) participant showing four dipoles (location and direction of electrical current) in associated brain regions in frontal, central, temporal, parietal, as well as occipital areas.** The signal magnitude reflects the estimated source activity.

Time-frequency analyses were performed in brain space using multiple source dipoles that modeled the main brain regions of interest (see **Figure 3**). The wide distribution of focal brain activity at scalp surfaces due to the nature of dipole fields and the smearing effect of volume conduction in EEG, means that scalp waveforms have mixed contributions from underlying brain sources, and thus measuring oscillatory activity on scalp surface electrodes may not be ideal. Optimal separation of brain activity was therefore achieved using source montages derived from a multiple source model where source waveforms separated different brain activities (see Scherg and Berg, 1991). The regional sources model used covered frontal, central, temporal, and parietal areas, as well as occipital areas. These sources are believed to be active in the processing of sensory-motor actions in our experiment (Zeki et al., 1991; Probst et al., 1993). In analyzing these sources, a 4-shell ellipsoidal head model (Berg and Scherg, 1994; Hoechstetter et al., 2004) was created for each participant and the source dipoles were inserted while the artifact-corrected coordinate files were appended.

**FIGURE 4:**
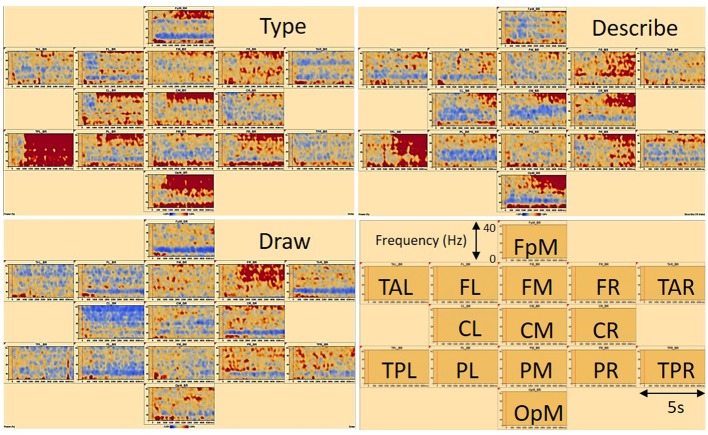
**Time-frequency displays of a typical (female) participant showing associated brain regions in frontal, temporal, central, parietal, and occipital areas of the brain.** FpM, fronto-polar midline; FL, frontal left; FM, frontal midline; FR, frontal right; TAL, temporal anterior left; TAR, temporal anterior right; TPL, temporal posterior left; TPR, temporal posterior right; CL, central left; CM, central midline; CR, central right; PL, parietal left; PM, parietal midline; PR, parietal right; OpM, occipito-polar midline. The signal magnitude on the y-axes (Power %) reflects the estimated neural activity in the various brain regions during the experimental conditions type, describe, and draw compared to baseline (–300 to 0 ms) activity. On the x-axes, baseline activity and 5 s recording time are displayed. Note that red areas indicate synchronization (ERS) and blue areas indicate desynchronization (ERD) of associated brain activity.

Time-frequency displays (see **Figure 4**), representing the change in amplitude over time [temporal spectral evolution (TSE)], were generated from the single trials by averaging spectral density amplitudes over trials. In this way, each graph displays the spectral amplitude density of one montage channel over time and frequency normalized to the baseline for each frequency (Pfurtscheller et al., 1994, 1996; Hoechstetter et al., 2004). To focus only on induced oscillatory brain activity, average evoked response signals were removed from the single trial time series before computing a TSE. Comparisons between the three conditions type, describe, and draw were computed for each participant. TSE displays were limited between frequency cut-offs of 4–40 Hz, while frequency and time sampling were set at 1 Hz, 40 ms.

A separate statistical program (BESA statistics 2.0^3^) was used to test the probability of significance in amplitude values and frequency ranges between each of the three experimental conditions in the TSE data for all participants. An average of TSE statistics for each participant could then be computed such that significant time-frequency ranges could be used as a guide in finding maximum oscillatory activities in each individual TSE. A combination of permutation tests and data clustering (see Ernst, 2004; Maris and Oostenveld, 2007) was employed in the statistical tests to address the multiple comparisons problem. Here, data clusters that showed a significant effect between conditions were assigned initial cluster values that were the sum of all *t*-values of all data points in each cluster. Using a paired *t*-test, these initial cluster values were passed through permutation and assigned new cluster values, such that the significance of the initial clusters could then be determined based on the distribution of the calculated cluster values assigned to each initial cluster after permutation. Cluster alpha (the significance level for building clusters in time and/or frequency) was set at 0.005, number of permutations (determined randomly without repetition) at 10,000, and frequency cut-offs kept the same as stated above, with epochs set from -300 to 5000 ms. Further statistical comparison of TSEs between our three experimental conditions for all participants was performed to compute probability maps to test for significant differences in the TSEs when comparing conditions (see **Figure 4**). Here, Bonferroni procedure and permutation tests as described by Simes (1986) and Auranen (2002) were used and applied to each set of time samples belonging to one frequency bin so as to correct for multiple testing. Frequency cut-offs and sampling points were maintained as stated above.

## Results

### Time-Frequency Responses

**Figure 4** displays the results of the TSE maps from a typical participant across brain regions of interest for the three experimental conditions type, describe, and draw. Brain regions of interest were located in frontal, temporal, central, parietal, and occipital areas of the brain. The signal magnitude (Power %) reflects the estimated neural activity in the various brain regions compared to baseline (-300 to 0 ms) activity. Increased spectral amplitude [induced synchronized activity, event related synchronization (ERS)] is shown as red colored contours (more dominant in the type and describe conditions) with decreased spectral amplitude [induced desynchronized activity, event related desynchronization (ERD)] shown as blue colored contours (more dominant in the draw condition).

**FIGURE 5:**
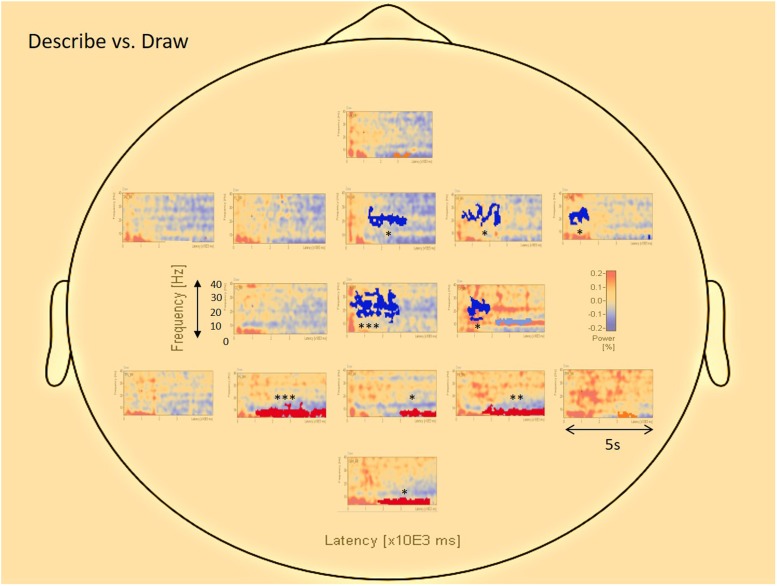
**The average visualization of significant (^∗∗∗^*p* < 0.0005; ^∗∗^*p* < 0.005; ^∗^*p* < 0.05) data clusters in the various sources of interest when the describe condition is compared to the draw condition.** Center and (right) frontal areas represent pre-motor, motor, and areas of creativity, whereas parietal and occipital areas represent sensory-motor integration and visual interpretation. Blue colors represent negative clusters, while red colors represent positive clusters. Each area in the central and frontal region is dominated by activity in the upper alpha (10–13 Hz), beta (12–20 Hz), and gamma (20–34 Hz) range, especially during the early parts of cognitive processing (ideation phase). Areas in the parietal and occipital region are dominated by activity in the theta (3–8 Hz) and alpha (8–13 Hz) range, almost for the entire drawing duration of the trials (execution phase).

**Figure 5** displays the differences in results of the permutation tests for the average of all participants between the conditions describe and draw. Only the differences between describe and draw are reported here because there were no clear differences found between type and describe. The permutation results (of clusters where the null hypothesis is rejected, i.e., data are not interchangeable) showed five significant negative clusters (in blue), in the central and right-frontal areas. The permutation results also showed four significant positive clusters (in red), in the parietal and occipital areas. Blue areas in the right frontal and central areas appeared to be dominated by activity in the upper alpha (10–13 Hz), beta (12–20 Hz), and gamma (20–34 Hz) range that was more prevalent during the earlier (ideation) parts of cognitive processing. Red areas in the parietal and occipital areas appeared to be dominated by activity in the theta (3–8 Hz) and alpha (8–13 Hz) range that was more prevalent during the execution stage of cognitive processing (see also **Table 1** for details).

**Table 1:** Permutation test results for nine significant clusters in decreasing order.

Cluster ID	*p*-value	Cluster value	Mean for describe	Mean for draw	Start time	End time	Start frequency	End frequency
CM	0.00023	-1763	-0.29	0.01	200	2900	11	36
PL	0.00032	1699	0.25	-0.25	1050	5000	4	16
PR	0.00385	1103	0.34	-0.27	1500	5000	4	13
OpM	0.00826	902	0.38	-0.26	1700	4600	4	9
FM	0.01380	-785	-0.29	0.02	1250	3400	16	30
FR	0.01402	-781	-0.27	0.06	400	2600	16	33
CR	0.02079	-687	-0.12	0.35	600	1850	13	31
PM	0.03831	537	0.45	-0.28	2950	5000	4	10
TAR	0.04594	-509	-0.18	0.17	350	1450	16	29

*CM, central midline; PL, parietal left; PR, parietal right; OpM, occipito-polar midline; FM, frontal midline; FR, frontal right; CR, central right; PM, parietal midline; TAR, temporal anterior right.*

## Discussion

In this experiment, high-density EEG was used in young adults to study brain electrical activity as a function of typing, describing, and drawing visually presented Pictionary^TM^ words in an attempt to explain the differences underlying traditional (keyboard) typing versus modern (stylus technology) drawing. TSE analyses were performed to investigate whether there were differences in brain activity in participants when they were using a laptop tablet keyboard versus using a laptop tablet pen. Apart from the absence of an ideation phase in the type condition, confirming that typing the same word repeatedly involves only shallow processing and no creativity, no clear differences in brain activity between typing and describing words were detected in the analyses. Therefore, we decided to concentrate fully on investigating the differences between describing and drawing words. A direct comparison between these conditions is interesting because both include a similar ideation phase (thinking how to describe/draw the seen word) but a different execution phase (typing on a keyboard versus drawing on the tablet).

### Higher-Frequency Oscillations in the Frontal and Central Regions

Our results showed that the ideation phase was most prominent in the describe condition where high-frequency oscillations (upper alpha/beta/gamma) were present during the first 2–3 s of each trial. This activity may be associated with higher cognitive thought processes as to how to describe the seen word in the best possible way. Especially the right pre-frontal areas of the brain have been associated with creativity in other studies (Srinivasan, 2007), albeit mostly in alpha power (e.g., Schwab et al., 2014; Jaarsveld et al., 2015), and could explain high activity in those parts of the brain during the describe condition. Despite the fact that neuroscientific studies into the neural mechanisms underlying creativity seem somewhat inconsistent, evidence is accumulating that EEG power is especially sensitive to requirements related to creativity during the ideation phase of a task.

We should keep in mind, however, that the observed oscillations in the frontal and central areas were characterized by induced synchronized activity (ERS), i.e., increased synchrony within the neural network, probably indicating less active cortical areas with decreased excitability of the neurons (Pfurtscheller and Lopes da Silva, 1999). Namely, when groups of neurons display such coherent synchronized activity, an active processing of information is rather unlikely and the corresponding networks are interpreted to be in a deactivated state. Therefore, the actual contribution of the frontal areas showing high-frequency synchronized oscillations in our results remains unclear.

### Low-Frequency Oscillations in the Parietal and Occipital Regions

Our results further showed that the execution phase was more prominent in the draw condition where low-frequency oscillations (theta/alpha) in the parietal and occipital areas were present during almost the entire trial apart from the first second or so. This activity may be associated with visual processing of the seen words and the subsequent sensory-motor integration during the entire stage of cognitive processing. Moreover, the activity present in the parietal and occipital areas also included induced desynchronized activity (ERD) within the associated neural networks, involving a decrease of spectral peak and amplitude attenuation, resulting in higher activation of cortical areas and increased excitability of the involved neurons. Such induced desynchronization is often taken to be an electrophysiological correlate of activated cortical areas involved in the processing of perceptual or cognitive information, or in the production of motor behavior (Pfurtscheller and Lopes da Silva, 1999). The more widespread and increased desynchronization found in the present study could indicate that a larger neural network is involved in information processing when drawing as opposed to describing the words, thereby facilitating and optimizing learning (Pfurtscheller, 1992). Proposed contributing factors to such improved desynchronization are: (1) increased task complexity (Vilhelmsen et al., 2015), (2) more efficient task performance (Boiten et al., 1992; Dujardin et al., 1993; Klimesch et al., 1996; Sterman et al., 1996; Agyei et al., 2015), and/or (3) more effort and attention as needed in patients and preterm infants (Agyei et al., 2016), the elderly, or participants with a lower IQ (Derambure et al., 1993; Neubauer et al., 1995; Defebvre et al., 1996; Neubauer et al., 1999).

Thus, desynchronized activity (ERD) in the parietal and occipital areas of the brain may have its beneficial effects on learning, particularly when it was shown to occur in the rather deep structures of the brain (c.f., red dipole, **Figure 3**) close to the limbic system, including the hippocampus, a brain area traditionally known for its association with learning.

Furthermore, recent studies suggest that theta-band oscillation and desynchronization (ERD), as shown in our results, may also be involved in mechanisms underlying sensory-motor integration (Bland and Oddie, 2001). Thus, because of its rich sensory-motor nature the involvement of drawing may have a beneficial effect on the learning process in general. Therefore, rich sensory-motor experiences seem to facilitate learning. In general, rich learning experiences will combine images that include shape patterns (occipital), tones and words (temporal and frontal), emotional connections (from the limbic system), and not the least movements (sensory-motor areas and the cerebellum) (Basar, 2004). Whenever movements are included as part of learning, more of the brain gets stimulated, resulting in the formation of more complex neural networks.

Thus, it seems that keyboards and pens bring into play different underlying neurological processes. This may not be surprising since handwriting/drawing is a complex task that requires the integration of various skills. Children, for example, take several years to master this precise skill. They have to learn how to hold the pen firmly while producing a different print for each letter. Operating a keyboard is something completely different since all one has to do is press the right key, and the typing movement is the same whatever the letter. Unlike typing, handwriting is the result of a unique sensory-motor movement of the body. Moreover, it can be argued that drawing each letter by hand also improves recognition. A study carried out by Longcamp and Velay (2005) showed that children aged three to five were better at recognizing letters when they learned to write the letters by hand as opposed to writing them on the keyboard.

## Conclusion

Mueller and Oppenheimer (2014) found evidence that lecture notes written in longhand were superior to verbatim keyboard notetaking with respect to learning outcome. Although the present study design did not allow us to test for recall and retention, we found direct electrophysiological evidence that drawing by hand activates larger networks in the brain than typing on a keyboard. When drawing target words using the laptop tablet stylus, relevant brain areas (parietal/occipital) showed desynchronized activity (ERD) in the theta/alpha range. Existing literature suggests that such oscillatory activity provides optimal conditions in the brain for learning. From the longhand notetaking findings together with the present results, a clear recommendation might be to combine traditional handwritten notes with visualizations (e.g., drawings, shapes, arrows, symbols) to facilitate and optimize learning. Sensory-motor information for the control of (pen) movement is picked up via the senses, and because of the involvement of the senses they leave a wider mark on establishing pathways in the brain, resulting in neural activity that governs all higher levels of cognitive processing and learning.

## Ethics Statement

This study was carried out in accordance with the recommendations of the Regional Ethics Committee (Central Norway) with written informed consent from all subjects. All subjects gave written informed consent in accordance with the Declaration of Helsinki.

## Author Contributions

AM and FW have contributed equally to the conception, design, analysis, and write-up of the work and are accountable for all aspects of the work.

## Conflict of Interest Statement

The authors declare that the research was conducted in the absence of any commercial or financial relationships that could be construed as a potential conflict of interest. Microsoft (Europe) provided the laptop tablets for the research.

## Footnotes

1 http://www.projectorcentral.com/ASK-M2.htm

2 https://www.pstnet.com/eprime.cfm

3 https://www.thegamegal.com

4 https://www.microsoft.com

5 https://www.egi.com/

6 http://www.besa.de/
